# Intra-urban differentials of fetal mortality in clusters of social vulnerability in São Paulo Municipality, Brazil

**DOI:** 10.1038/s41598-021-03646-5

**Published:** 2021-12-20

**Authors:** Lays Janaina Prazeres Marques, Zilda Pereira da Silva, Bárbara Laisa Alves Moura, Rossana Pulcineli Vieira Francisco, Marcia Furquim de Almeida

**Affiliations:** 1 Department of Epidemiology, School of Public Health of the University of São Paulo, Av. Dr. Arnaldo, 715 - Cerqueira César, São Paulo, SP 01246-904 Brazil; 2 Department of Obstetrics and Gynecology, School of Medicine of the University of São Paulo, São Paulo, Brazil

**Keywords:** Medical research, Epidemiology

## Abstract

This study aimed to analyze the distribution of stillbirths by birth weight, type of death, the trend of Stillbirth Rate (SBR), and avoidable causes of death, according to social vulnerability clusters in São Paulo Municipality, 2007–2017. Social vulnerability clusters were created with the k-means method. The Prais-Winsten generalized linear regression was used in the trend of SBR by < 2500 g,  ≥ 2500 g, and total deaths analysis. The Brazilian list of avoidable causes of death was adapted for stillbirths. There was a predominance of antepartum stillbirths (70%). There was an increase in SBR with the growth of social vulnerability from the center to the outskirts of the city. The cluster with the highest vulnerability presented SBR 69% higher than the cluster with the lowest vulnerability. SBR ≥ 2500 g was decreasing in the clusters with the high vulnerability. There was an increase in SBR of avoidable causes of death of the cluster from the lowest to the highest vulnerability. Ill-defined causes of death accounted for 75% of deaths in the highest vulnerability area. Rates of fetal mortality and avoidable causes of death increased with social vulnerability. The trend of reduction of SBR ≥ 2500 g may suggest improvement in prenatal care in areas of higher vulnerability.

## Introduction

Stillbirth is one of the most common adverse perinatal outcomes worldwide, constituting a relevant indicator of prenatal and childbirth care. It is estimated that 2.6 million stillbirths occur per year in the third trimester of pregnancy. Prevention of these deaths represents a great challenge, especially due to the disparities in their occurrence between countries and within them, especially in those of low and middle income, because they concentrate 98% of deaths^1,2^.

There is a wide variation in the stillbirth rate (SBR), with an unequal reduction in the world, being slower in low and middle-income countries, with values 10 times higher when compared to high-income countries^2,3^. Between 2000 and 2015, there was a 25.5% decline in the global SBR, from 24.7 to 18.4 fetal deaths per thousand births, respectively^4^. However, in Latin America and the Caribbean, an increase from 5.9 to 6.8 was observed between 2000 and 2016^5^. In the same period, SBR in Brazil showed a stationary trend, ranging from 4.9 to 5.8^5,6^.

It is estimated that 3/4 of fetal deaths can be prevented with adequate access to quality care and early detection of risky pregnancies^7^. Potentially avoidable stillbirths are considered “sentinel events” of care received. In Brazil, the analysis of these deaths is part of the routine investigation by the Surveillance of Infant and Fetal Death (*Vigilância do Óbito Infantil e Fetal*—VOIF)^8^, a practice equivalent to stillbirths auditing^9^. Birth weight is considered the isolated factor of the greatest importance for fetus survival, and birth weight greater than or equal to 2500 g is established as a potentially avoidable parameter^10^. In addition, the relevance of identifying avoidable causes of death for the analysis of SBR and the definition of areas that need priority attention to reduce avoidable deaths is highlighted^11^.

Interventions to prevent stillbirth go through the recognition of social inequities and spatial disparities in the distribution of stillbirths^12,13^. Given the heterogeneous context of São Paulo Municipality, represented by urban areas of high social inequality, more granular geographical levels and group non-neighboring areas with similar living conditions must be considered, even those located in different regions of the city. The use of a synthetic index that measures poverty, without restricting this condition to income deprivation and including the aspects that conform to it, such as family composition and educational level, can contribute to the understanding of avoidable stillbirths in vulnerable areas^14,15^.

Therefore, the goal of the present study was to analyze the distribution of stillbirths by birth weight, type of death, the trend of stillbirth rate, and avoidable causes of death, according to social vulnerability clusters in São Paulo Municipality, from 2007 to 2017.

## Methods

This is an ecological study and time series that included all stillbirths of mothers living and occurred in São Paulo Municipality (*Município de São Paulo*—MSP), between 2007 and 2017. The data collection unit refers to the 96 administrative districts of the MSP. The District of Marsilac was not included because it is a predominantly rural area.

Brazil is an urbanized middle-income country, being the largest country in South America and the fifth-largest in the world in terms of land area and sixth in population, with more than 213 million inhabitants^16^. About 22% of the Brazilian population is concentrated in the São Paulo State, which is located in the Southeast Region of Brazil, the most developed in the country^16^. São Paulo Municipality is the state capital and covers an area of 1509 km^2^. Its estimated population is 12.1 million inhabitants in 2017, representing the largest urban center in Latin America. The city's average per capita income is USD 865.38 and the Municipal Human Development Index is 0.805^16^.

The data comes from the Mortality Information System (*Sistema de Informações sobre Mortalidade*—SIM) of Brazil and were provided by the Mortality Information Enhancement Program (*Programa de Aprimoramento das Informações de Mortalidade*—PRO-AIM) of the Epidemiology and Information Coordination (*Coordenação de Epidemiologia e Informação*—CEInfo) of the Municipal Health Department of São Paulo. Access to information is also available through at: www.prefeitura.sp.gov.br/tabnet.

The definition of stillbirth adopted was those which occurred from the 22nd week of pregnancy or had birth weight ≥ 500 g. To compose the denominator of SBR (stillbirths divided by the sum of live births and stillbirths), live births data from the System of Information on Live Births (*Sistema de Informações Sobre Nascidos Vivos*—Sinasc) were used.

Clusters of districts were identified to analyze the spatial distribution of deaths. The ratio of the population classified by the São Paulo Social Vulnerability Index (*Índice Paulista de Vulnerabilidade Social*—IPVS) of the State Data Analysis System Foundation was adopted^15^. The IPVS is an indicator composed of nine variables (five for the socioeconomic dimension and four for the demographic dimension): household income per capita, the average income of the woman heads of the household, the ratio of households with per capita income of up to 1/2 minimum wage(s) (MW) and up to 1/4 of the MW, percentage of heads of household < 30 years old, women head of household < 30 years old, the average age of heads of households, and percentage of children < 6 years old. From multivariate statistical techniques (factor analysis and cluster analysis) applied for these variables, six groups of vulnerability were obtained originally for MSP: 1) lowest vulnerability; 2) very low; 3) low; 4) medium; 5) high (urban), and 6) very high (slums)^15^.

The population of each district can be classified into six vulnerability categories. This means that a district is not classified into a single vulnerability category due to the heterogeneity of its population. Based on the similarity of population distribution in the IPVS categories, the k-means method was applied to group the districts. The definition of the number of clusters was based on the graphical analysis of the dendrogram, with three clusters being obtained. ArcGIS software version 10.3 (https://www.arcgis.com) was used to observe the geographic composition and the distribution of average SBR for the period according to social vulnerability clusters.

Information on birth weight (< 2500 g and ≥ 2500 g) was obtained from Death Certificates (DC). The significance of the weight differences between the clusters was verified by the Pearson chi-square test (χ^2^) (*p* < 0.05). The type of death was identified with the variable “Death in relation to the birth”, grouped under the following: antepartum, intrapartum, and unknown (without information and ignored). Such information was integrated as one of the main points of the new approach to the Classification of Perinatal Mortality (ICD-PM) of the World Health Organization (WHO), which presumes the identification of the type of death (antepartum, intrapartum, and unknown)^17^.

Prais-Winsten generalized linear regression was used in the time series analysis. The dependent variable was the logarithm of the SBR and the independent was calendar years. The presence of serial autocorrelation was evaluated with the Durbin-Watson statistic (*d*). The lower (dI = 0.653) and higher (dS = 1.010) critical values were compared, obtained from sample size (*n* = 11) and regressors, excluding the intercept (*k*’ = 1). If *d* > *dS*, there is evidence of first-order positive autocorrelation (ρ > 0). Three models were obtained for each stratum: < 2500 g;  ≥ 2500 g; and total deaths. The Annual Percent Change (APC) was obtained by^18^: $APC=\left[-1+{10}^{{\beta }_{1}}\right]\bullet 100$ and the respective 95% confidence intervals: $95\mathrm{\%CI}=\left[-1+{10}^{{\beta }_{1min}}\right]\bullet 100;\left[-1+{10}^{{\beta }_{1max}}\right]\bullet 100$. It was verified whether the trend of the rates was stationary (*p* ≥ 0.05), decreasing (*p* < 0.05 and *β*_1_ negative), or increasing (*p* < 0.05 and β_1_ positive). P-values were obtained with the Wald test. The significance level adopted in the study was 5%. In the graphical analysis of the time series, the smoothing technique by moving averages was used, to the detriment of white noise from the small number of deaths. The tabs and analyses were created using Excel® (https://products.office.com/) and Stata 13 software (*StataCorp. 2013. Stata Statistical Software: Release 13. College Station, TX: StataCorp LP*—https://www.stata.com).

**Figure 1:**
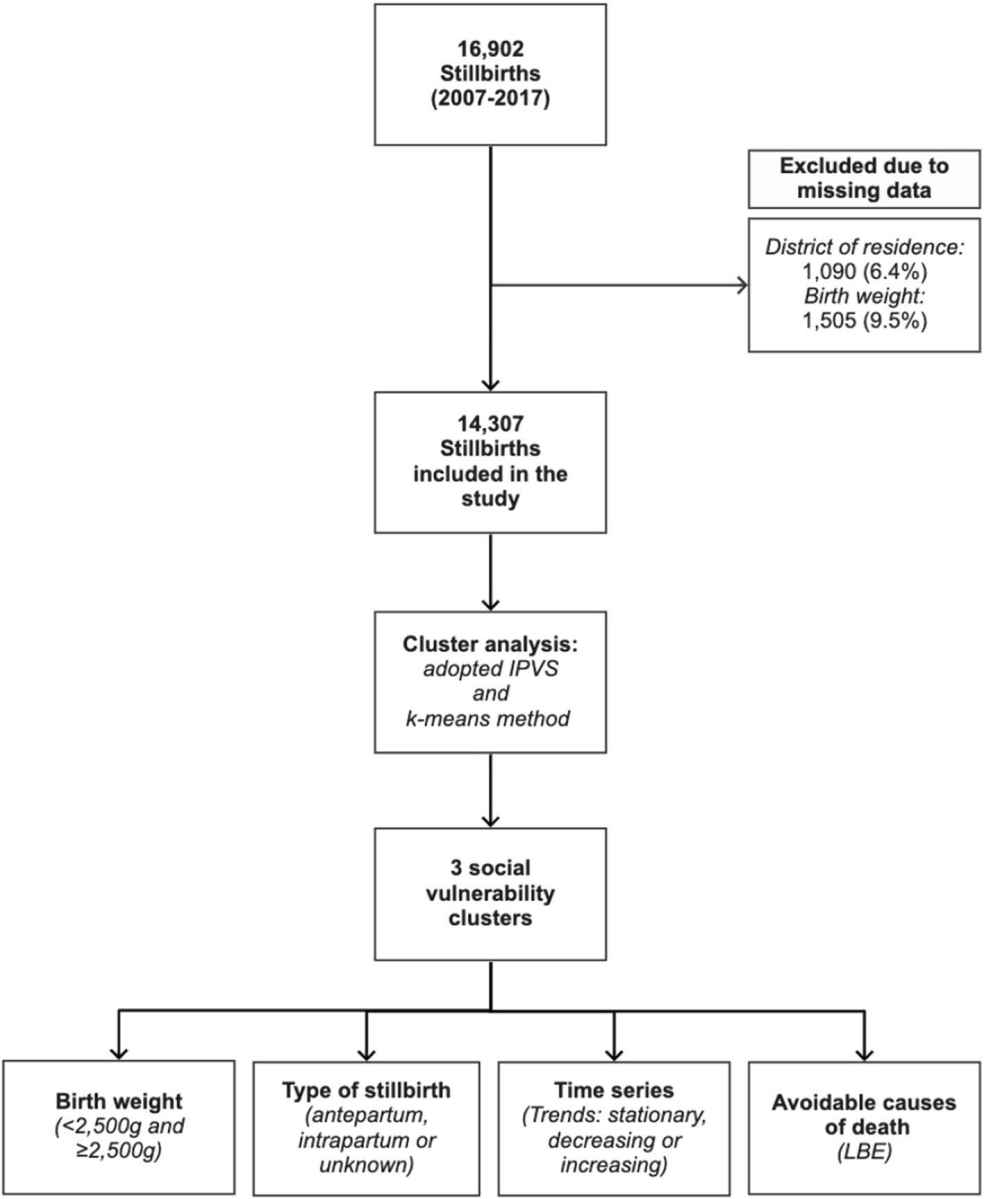
Flowchart of the steps of the study.

The Brazilian List of Avoidable Causes of Death (*Lista Brasileira de Causas de Mortes Evitáveis*—LBE) was adopted, which organizes deaths by groups of underlying cause of death, according to the International Statistical Classification of Diseases and Related Health Problems 10^th^ Revision (ICD-10). LBE is divided into three groups: avoidable causes, ill-defined causes, and other causes (not clearly avoidable). In the avoidable causes group, deaths are also classified according to subgroups of healthcare actions: immunoprevention; adequate care for women during pregnancy; adequate care for women in childbirth; adequate care for the fetus and newborn; adequate diagnostic and treatment; and adequate health promotion actions, related to adequate healthcare actions^19^. Adjustments were made for the use of LBE for stillbirths, since it was initially developed for infant deaths and some codes, groups, and morbidity conditions are specific to the newborn and do not cover stillbirths (Supplementary Table S1). Figure 1 shows the flowchart of the steps of the study.

This is research with public data, eliminating the need to obtain informed consent from all participants, since the database is available electronically. Additionally, all methods were carried out by relevant guidelines and regulations. The project of research was approved by the Research Ethics Committee of the School of Public Health of the University of São Paulo under protocol number 3.215.709 in 2019.

## Results

During the study period, there were 16,902 stillbirths, of which 1090 (6.4%) had no specification of the mother's district of residence and another 1505 (9.5%) lacked information on birth weight, being excluded. Thus, 14,307 stillbirths were included in the study.

**Figure 2:**
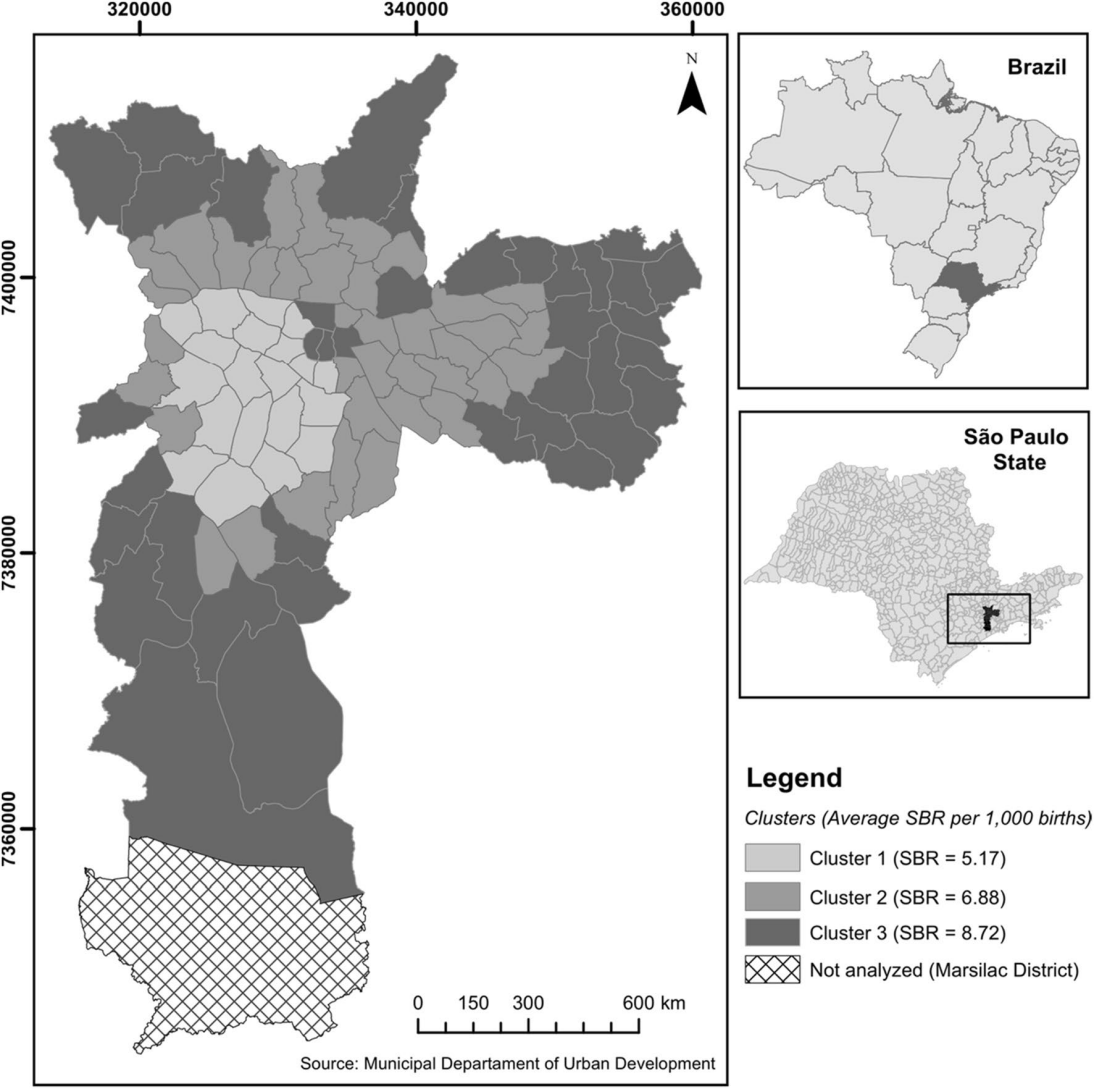
Average Stillbirth Rate (per thousand births) according to clusters of social vulnerability, São Paulo Municipality, 2007–2017. *Note:* SBR—stillbirth rate. **Cluster 1** (Alto de Pinheiros, Barra Funda, Bela Vista, Butantã, Campo Belo, Consolação, Itaim Bibi, Jardim Paulista, Lapa, Liberdade, Moema, Morumbi, Perdizes, Pinheiros, Santa Cecília, Santo Amaro, Saúde, Vila Andrade, Vila Leopoldina, and Vila Mariana); **Cluster 2** (Água Rasa, Aricanduva, Artur Alvim, Belém, Cachoeirinha, Cambuci, Campo Grande, Carrão, Casa Verde, Cidade Líder, Cursino, Freguesia do Ó, Ipiranga, Jabaquara, Jaguara, Jaguaré, Limão, Mandaqui, Mooca, Pari, Penha, Pirituba, Ponte Rasa, Rio Pequeno, Sacomã, Santana, São Domingos, São Lucas, Socorro, Tatuapé, Tucuruvi, Vila Formosa, Vila Guilherme, Vila Matilde, Vila Medeiros, Vila Prudente, and Vila Sônia); **Cluster 3** (Anhanguera, Bom Retiro, Brás, Brasilândia, Campo Limpo, Cangaiba, Capão Redondo, Cidade Ademar, Cidade Dutra, Cidade Tiradentes, Ermelino Matarazzo, Grajaú, Guaianases, Iguatemi, Itaim Paulista, Itaquera, Jaraguá, Jardim Ângela, Jardim Helena, Jardim São Luís, Jaçanã, José Bonifácio, Lajeado, Parelheiros, Parque do Carmo, Pedreira, Perus, Raposo Tavares, República, São Mateus, São Miguel, São Rafael, Sapopemba, Sé, Tremembé, Vila Curuçá, Vila Jacuí, and Vila Maria).

Three clusters of social vulnerability were obtained, which were composed of 20 administrative districts in cluster 1; 37 in cluster 2; and 38 in cluster 3. Cluster 1 is a predominantly rich area, with better living conditions and very low social vulnerability, representing 14% of the population. Cluster 2 encompasses expressive portions of the middle or transitional classes, and includes 32.5% of MSP residents. Cluster 3 is represented by the majority of residents (53.5%) and is located on the outskirts of the city, where there is a concentration of slums, considered vulnerable to poverty. Cluster 1 accounted for 7.0% of stillbirths occurring in MSP; cluster 2 concentrated 27.2% of them; whereas cluster 3 presented more than half of deaths (65.8%). From 2007 to 2017, the average annual stillbirth rate in MSP was 7.78 fetal deaths per thousand births. The average SBR in cluster 3 was 69% higher about cluster 1 and 27% higher about cluster 2, configuring a gradient of increase in mortality from the least to the most vulnerable area, with spatial distribution from the center to the periphery (Fig. 2).

**Figure 3:**
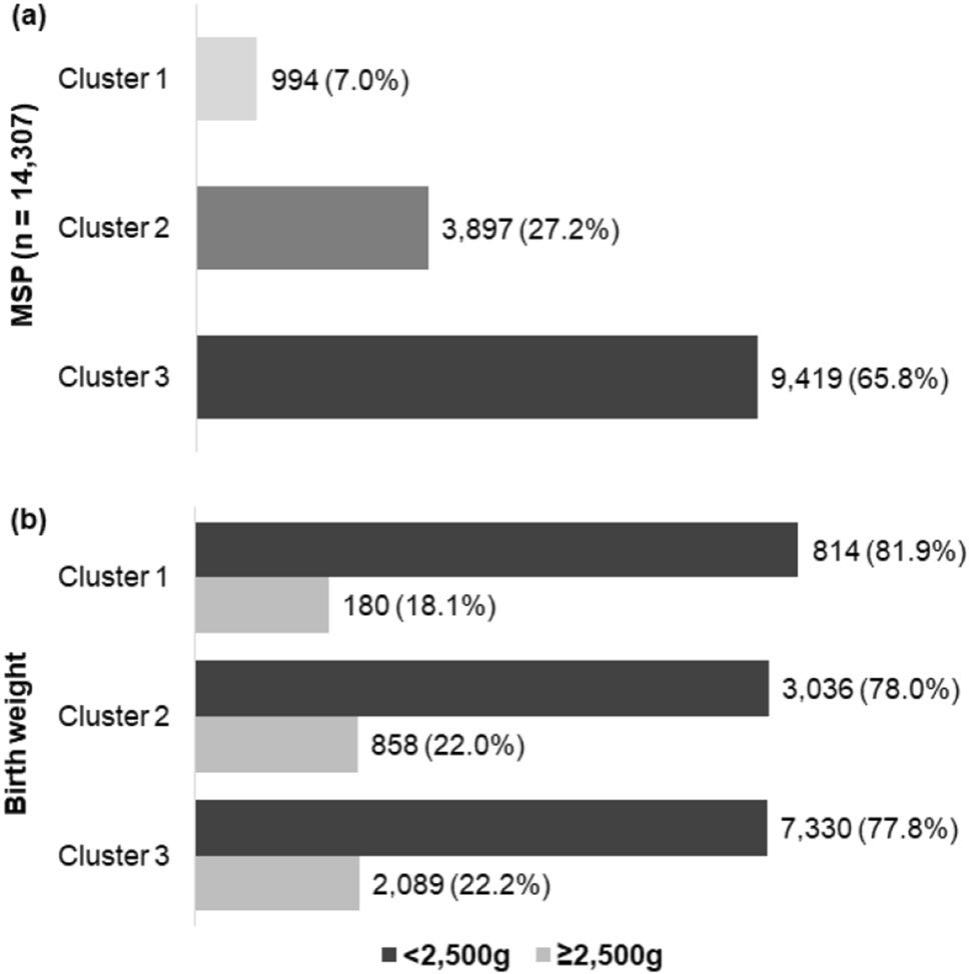
Distribution of stillbirths by clusters of social vulnerability and birth weight, São Paulo Municipality, 2007–2017. (**a**) Cluster distribution in MSP. (**b**) Distribution by clusters and birth weight (< 2500 g and ≥ 2500 g).

Of the 14,307 stillbirths, 78.1% (n = 11,180) weighed < 2500 g, whereas 21.9% (n = 3127) presented ≥ 2500 g (supplementary Fig. S1). The ratio of deaths with ≥ 2500 g, considered potentially avoidable, showed a significant trend of growth with the increase in vulnerability (*p* < 0.05) (Fig. 3).

Antepartum deaths predominated in all clusters and birth weight groups. However, there is a considerable ratio of deaths of unknown type, as well as an increase in this proportion from the center to the peripheral areas, accounting for about 30% of the total deaths in cluster 3. Cluster 1 presented a higher proportion of antepartum and intrapartum deaths among birth weight groups. However, SBR of intrapartum deaths < 2500 g was 50% higher in the city’s outskirts about downtown (Table 1).

**Table 1:** Number of stillbirths and stillbirth rate (per thousand births), total and by birth weight, according to the type of death and clusters of social vulnerability, São Paulo Municipality, 2007–2017.

Type of death	Cluster 1			Cluster 2			Cluster 3		
	n	%	SBR	n	%	SBR	n	%	SBR
** < 2500 g**									
Antepartum	635	78.01	33.65	2265	74.60	40.93	5141	70.14	47.01
Intrapartum	15	1.84	0.79	50	1.65	0.90	127	1.73	1.16
Unknown	164	20.15	8.69	721	23.75	13.03	2062	28.13	18.85
Total	814	100.00	43.14	3036	100.00	54.87	7330	100.00	67.02
** ≥ 2500 g**									
Antepartum	134	74.44	0.77	636	74.13	1.25	1,442	69.03	1.49
Intrapartum	4	2.22	0.02	11	1.28	0.02	33	1.58	0.03
Unknown	42	23.33	0.24	211	24.59	0.41	614	29.39	0.63
Total	180	100.00	1.04	858	100.00	1.68	2,089	100.00	2.15
**Total deaths**									
Antepartum	769	77.36	4.00	2901	74.50	5.12	6,583	69.89	6.09
Intrapartum	19	1.91	0.10	61	1.57	0.11	160	1.70	0.15
Unknown	206	20.72	1.07	932	23.93	1.65	2676	28.41	2.48
Total	994	100.00	5.17	3894	100.00	6.88	9419	100.00	8.72

Note: SBR—stillbirth rate.

The three clusters showed a stationary trend of SBR in total deaths and those with birth weight < 2500 g (*p* ≥ 0.05). Among deaths of that > 2500 g, there was a decreasing trend in clusters 2 and 3 (− 0.7% and − 1.8% per year, respectively) and a stationary trend in cluster 1 (*p* ≥ 0.05). The annual decrease percentage was more pronounced in cluster 3. There was no serial autocorrelation in all models (Table 2). Despite the decreasing trend of the SBR of potentially avoidable deaths (≥ 2500 g), cluster 3 presented a rate similar to cluster 2 in 2017, which was 125% higher than that of cluster 1. Supplementary Fig. S2 shows the annual trend, according to weight groups and total deaths.

**Table 2:** Estimates of the Prais-Winsten regression for the total stillbirth rate (per thousand births) and by birth weight, according to clusters of social vulnerability, São Paulo Municipality, 2007–2017.

Cluster	Annual stillbirth rate											APC^a^	_95%_CI	Trend
	2007	2008	2009	2010	2011	2012	2013	2014	2015	2016	2017			
** < 2500 g**														
Cluster 1	40.6	50.1	31.6	35.9	41.7	42.9	47.9	45.4	46.1	50.7	43.0	2.0%	− 0.71; 4.77	Stationary
Cluster 2	48.2	53.1	55.7	47.2	55.0	54.7	60.0	62.3	57.1	57.4	52.9	1.4%	− 0.22; 2.99	Stationary
Cluster 3	60.4	70.4	66.0	64.5	60.5	64.9	71.3	70.6	69.0	69.2	70.3	1.1%	− 0.09; 2.26	Stationary
** ≥ 2500 g**														
Cluster 1	1.4	1.2	1.1	0.9	0.7	1.4	0.9	0.9	0.8	1.3	0.8	− 2.0%	− 5.31; 1.43	Stationary
Cluster 2	1.9	1.6	1.9	1.5	1.7	1.6	1.8	1.5	1.7	1.6	1.7	− 0.7%	− 1.38; − 0.11	Decreasing^b^
Cluster 3	2.3	2.6	2.2	2.3	2.3	1.8	2.1	1.9	2.2	2.2	1.8	− 1.8%	− 3.40; 0.24	Decreasing^b^
**Total death**														
Cluster 1	5.2	5.9	4.1	4.5	4.7	5.7	5.5	5.2	5.2	5.8	5.1	0.9%	− 1.52; 3.41	Stationary
Cluster 2	6.4	6.6	7.4	5.9	6.8	6.9	7.6	7.3	7.0	7.2	6.7	0.9%	− 0.30; 2.15	Stationary
Cluster 3	8.1	9.3	8.7	8.6	8.2	8.2	9.2	8.9	8.9	9.1	8.8	0.5%	− 0.43; 1.46	Stationary

^Note: a^APC—annual percentage change; ^b^*p* < 0.05 (Wald Test).

Regarding the avoidable causes of death with < 2500 g, an increased gradient of SBR in the downtown-outskirt direction was identified in all deaths. Cluster 1 presented a higher proportion of avoidable causes and a lower proportion of ill-defined causes in all groups of deaths. However, cluster 1 obtained the highest proportion of non-clearly avoidable causes, which refer to congenital malformations, when compared to the other clusters in almost all deaths (Supplementary Table S2 and S3). The ill-defined causes of death represented more than 80% of the total deaths in the three clusters of deaths of unknown type, regardless of birth weight. Despite the small number of intrapartum deaths with ≥ 2500 g, avoidable deaths were more frequent in cluster 3 (60.6%) (Table 3).

**Table 3:** Number of stillbirths and stillbirth rate (per thousand births), according to the Brazilian List of Avoidable Causes of Death (LBE), by birth weight and clusters of social vulnerability, São Paulo Municipality, 2007–2017.

LBE groups and type of death	Cluster 1			Cluster 2			Cluster 3		
<2500g									
** Antepartum**	n = 635	%	SBR	n = 2,265	%	SBR	n = 5,141	%	SBR
**Avoidable causes of death**	165	25.98	8.74	474	20.93	8.57	996	19.37	9.11
Reducible with adequate healthcare to women and their fetus during pregnancy	165	25.98	8.74	474	20.93	8.57	996	19.37	9.11
**Ill-defined causes of death**	361	56.85	19.13	1,594	70.38	28.81	3828	74.46	35.00
**Other causes (not clearly avoidable)**	109	17.17	5.78	197	8.70	3.56	317	6.17	2.90

Intrapartum	n = 15	%	SBR	n = 50	%	SBR	n = 127	%	SBR
**Avoidable causes of death**	10	66.67	0.53	25	50.00	0.45	67	52.76	0.61
Reducible with adequate care to women and their fetus during pregnancy	6	40.00	0.32	15	30.00	0.27	47	37.01	0.43
Reducible by adequate healthcare to women in childbirth	4	26.67	0.21	10	20.00	0.18	20	15.75	0.18
**Ill-defined causes of death**	3	20.00	0.16	17	34.00	0.31	43	33.86	0.39
**Other causes (not clearly avoidable)**	2	13.33	0,11	8	16.00	0,14	17	13.39	0.16

Unknown	n = 164	%	SBR	n = 721	%	SBR	n = 2,062	%	SBR
**Avoidable causes of death**	15	9.15	0.79	49	6.80	0.89	140	6.79	1.28
Reducible with adequate healthcare to women and their fetus during pregnancy	9	5.49	0.48	36	4.99	0.65	106	5.14	0.97
Reducible by adequate healthcare to women in childbirth	6	3.66	0.32	13	1.80	0.23	34	1.65	0.31
**Ill-defined causes of death**	139	84.76	7.37	634	87.93	11.46	1,838	89.14	16.81
**Other causes (not clearly avoidable)**	10	6.10	0.53	38	5.27	0.69	84	4.07	0.77

≥ 2500 g									
** Antepartum**	n = 134	%	SBR	n = 636	%	SBR	n = 1,442	%	SBR
**Avoidable causes of death**	38	28.36	0.22	135	21.23	0.26	227	15.74	0.23
Reducible with adequate healthcare to women and their fetus during pregnancy	38	28.36	0.22	135	21.23	0.26	227	15.74	0.23
**Ill-defined causes of death**	89	66.42	0.51	475	74.69	0.93	1,166	80.86	1.20
**Other causes (not clearly avoidable)**	7	5.22	0.04	26	4.09	0.05	49	3.40	0.05

Intrapartum	n = 4	%	SBR	n = 11	%	SBR	n = 33	%	SBR
**Avoidable causes of death**	2	50.00	0.01	2	18.18	0.00	20	60.61	0.02
Reducible with adequate healthcare to women and their fetus during pregnancy	1	25.00	0.01	2	18.18	0.00	8	24.24	0.01
Reducible by adequate attention to women in childbirth	1	25.00	0.01	–	–	–	12	36.36	0.01
**Ill-defined causes of death**	1	25.00	0.01	8	72.73	0.02	12	36.36	0.01
**Other causes (not clearly avoidable)**	1	25.00	0.01	1	9.09	0.00	1	3.03	0.00

Unknown	n = 42	%	SBR	n = 211	%	SBR	n = 614	%	SBR
**Avoidable causes of death**	3	7.14	0.00	13	6.16	0.03	29	4.72	0.03
Reducible with adequate healthcare to women and their fetus during pregnancy	1	2.38	0.00	3	1.42	0.01	14	2.28	0.01
Reducible by adequate attention to women in childbirth	2	4.76	0.00	10	4.74	0.02	15	2.44	0.02
**Ill-defined causes of death**	37	88.10	0.02	192	91.00	0.38	560	91.21	0.58
**Other causes (not clearly avoidable)**	2	4.76	0.00	6	2.84	0.01	25	4.07	0.03

Note: SBR—stillbirth rate; LBE—Brazilian List of Avoidable Causes of Death (*Lista Brasileira de Causas de Mortes Evitáveis*).

## Discussion

Social vulnerability grew from downtown to the outskirts of MSP. An increase gradient in average SBR was identified, besides ratio of deaths with weight ≥ 2500 g, SBR due to avoidable causes, and worse quality of data record (ratio of deaths of unknown type and due to ill-defined causes of death) with the increase of social vulnerability. There was a steady trend in the SBR of total deaths and those with < 2500 g, and a decreasing trend in deaths with ≥ 2500 g in the clusters of medium and high vulnerability (clusters 2 and 3).

The average SBR in the period studied increased as social vulnerability increased. The results of SBR of MSP and all clusters are below the goal of reducing avoidable stillbirths, proposed by the Action Plan for Each Newborn, which estimates an SBR of less than 10 stillbirths per thousand births by 2035^20^. However, the average SBR in cluster 3 (9.59 deaths per thousand births) is higher than those found in 2015 for Brazil (8.6) and middle and high-income Latin countries, such as Argentina (4,6), Colombia (8.1), Cuba (6.2), Chile (3.1), Ecuador (7.7), Venezuela (7.1); and were lower than that of Paraguay (13.4), Bolivia (12.9), and Guatemala (11.9) when considering the WHO definition of stillbirths for international comparisons (≥ 28 weeks of pregnancy)^21^.

The regions of high social vulnerability (cluster 3) concentrated stillbirths and those with greater potential for prevention (≥ 2500 g). The clusters of social vulnerability express the existing interaction between individuals residing in these areas, who share socioeconomic and demographic characteristics, and similar living and health conditions. This conformation is represented by residential segregation, marked by the concentration of poverty in the outskirts of metropolitan regions, distant from urban centers^13^. Individual (extremes of age, low education, non-white ethnicity, presence of comorbidities, and unfavorable reproductive history) and behavioral (smoking, excessive alcohol consumption, and inadequate nutritional habits) factors present in deprived areas, combined with limited socioeconomic resources and insufficient prenatal care, contribute to greater vulnerability of mothers to risk situations that favor the occurrence of stillbirths^3,14^.

The predominant type of stillbirth in all clusters was antepartum, similar to what occurs in developed countries^21^ and Brazilian urban centers^22,23^. The small fraction of intrapartum deaths observed reflects trends in developed countries, where 10% of stillbirths occur during childbirth^2^. Antepartum stillbirth is a complex syndrome associated with sociodemographic characteristics, the presence of comorbidities, and conditions typical of pregnancy, amenable to treatment when identified early with adequate prenatal care^2,20^. On the other hand, intrapartum deaths may be related to the attention given at the time of delivery^2^.

The type of stillbirths could not be identified in more than 1/4 of the total. The lack of information increased with social vulnerability. Approximately all stillbirths occurred in hospitals^24^, and since fetal heart rate monitoring is part of the hospital routine, this should contribute to the proper recording of information^9^.

There was a steady trend of SBR in total deaths and those with birth weight < 2500 g, similar to what occurs in Brazil^6^. Potentially avoidable deaths (≥ 2500 g) showed a decreasing trend in clusters 2 and 3, following the declining trend in MSP (-1.3% per year)^24^ and Latin America (-2.0% per year)^21^. A study conducted in a city in the Southern region of Brazil pointed out a trend of reduction of SBR over three decades, with a sharp decline in the poorest income tertile^12^. The declining trend observed may be related to two aspects: the increase in access to prenatal care with the expansion of the coverage of the Family Health Strategy and the Primary Health Care network, located predominantly in the peripheral area of the city^12,25^. In this period, there was an increase in women’s schooling and a reduction in income inequalities, achieved with income transfer programs, such as the Brazilian Family Allowance Programme (*Bolsa Família*)^3,12^. In addition, the effectiveness of surveillance actions in the investigation of stillbirths with weight ≥ 2500 g can be added to these factors^24^.

Cluster 1 is the area of least vulnerability of MSP, where there was no significant reduction in SBR. A possible explanation for this fact may be the relatively low incidence of stillbirths, capable of weakening the strength of statistical associations^20^. However, this should be better explored by other researchers who identify the factors related to this outcome.

The stillbirth of unknown type showed 3/4 of ill-defined causes of death. The Brazilian List of Avoidable Causes was initially formulated for infant deaths, and adaptations were made for its use in stillbirths. The main change introduced was considering intrauterine hypoxia (P20) as an ill-defined cause. This syndrome has several origins and can be considered a “garbage code”, that is, it represents an indication of a non-specific cause of death, which hinders the implementation of measures for its prevention and indicates the absence of adequate information to define the underlying cause of death, and can also be used as an indicator of the quality of care provided^9,26^. However, this cause accounted for about 40% of all stillbirths. Thus, the proportion of ill-defined causes identified in this study is higher than those found in other studies that used LBE without this adaptation and maintained this cause in the “Reducible with adequate healthcare provided to women in childbirth” group^23,27–29^. This was also the main cause of antepartum deaths (44%), thus showing itself incongruous with its allocation as reducible with adequate healthcare provided during childbirth.

In addition, an increase in data incompleteness and ratio of ill-defined causes with increased vulnerability was found. In MSP, most stillbirths are referred to the Death Verification Service (DVS) to define the cause of death with necropsy. However, ill-defined causes constitute the main cause of death. This paradox can be attributed to the absence of referral of the placenta and the hospital’s records of pregnant women to the DVS and the presence of macerated fetuses^30^. A study conducted in MSP indicated that surveillance actions in the investigation of stillbirths contribute to the improvement of causes of mortality. However, 2/3 of the causes remained ill-defined after the investigation^24^. Research indicates that in low and middle-income countries, about half of stillbirths remain with an undetermined or ill-defined cause, mainly due to the absence of data and postmortem assessments^9,31^.

The area of least vulnerability (cluster 1) had the highest ratio of avoidable causes in almost all types of death, regardless of weight. This result is contradictory to what was expected. However, this may have been influenced by the lower proportion of ill-defined causes when compared to other clusters. Thus, a larger number of causes would be prone to classification in the subgroups of avoidable causes. This cluster also presented a higher proportion of non-clearly avoidable causes, among which congenital malformations. This profile is similar to that of a study conducted with data from 50 countries, which identified a lower ratio of ill-defined causes and a higher proportion of stillbirths due to congenital anomalies in high-income environments, which represent the most difficult causes to prevent^31^.

On the other hand, in all groups of deaths studied, there was an increased gradient in mortality rates due to avoidable causes of death with increased vulnerability. This rate is an indicator that reflects the access and quality of healthcare services and signals the existence of deficiencies in the healthcare provided^11^. This gradient is consistent with the result obtained in a capital in Northeastern Brazil, which also identified an increase in the rates of avoidable fetal mortality in regions marked by the presence of greater social deprivation^28^.

Preventing avoidable stillbirths requires a higher quality of data, especially the indication of the causes of death. More than 80 classification systems have been developed over decades. Most were incompatible with the general principles of the ICD-10, making regional comparisons impossible. Facing this difficulty, in 2016, the WHO launched a new approach to the classification of perinatal deaths: “application of the WHO ICD-10 to perinatal deaths” (ICD-perinatal mortality or ICD-PM). This system requires the registration of the type of death (intrapartum or antepartum) and maternal conditions that contributed to perinatal death^9,17^. Despite its relevance and application worldwide, the ICD-PM is not widely used to date and has not been implemented in Brazil^17,24^. The application of the ICD-PM can be promising in improving the indication of causes of death and in directing the prioritization of areas in which improvements in the quality of care will have a greater impact, since it uses the identification of antepartum and intrapartum deaths^9,32^.

The limitations of the present study refer to the use of secondary data, whose quality of some variables is poor, such as the underlying cause and the identification of the type of death. In addition, the unit of data analysis by aggregate districts comprises large geographical and population areas, thus not ensuring its homogeneity.

## Conclusions

Despite its relevance, fetal mortality is not part of the set of monitoring indicators in Brazil and was also not mentioned in the Brazilian National Health Plan, which defines priorities and aims to achieve goals by 2023. Obtaining improvements in the quality of information and reducing avoidable stillbirths, especially in the areas of high social vulnerability, which concentrate most deaths, raises the need for increased investments and prioritization of strategies that contribute to greater visibility of fetal mortality.

## Supplementary Information

Supplementary Information.
