# Supplementary material for: Intra-urban differentials of fetal mortality in clusters of social vulnerability in São Paulo Municipality, Brazil
Source: Sci Rep. 2021 Dec 20;11:24256. doi: 10.1038/s41598-021-03646-5 (PMC8688466; doi:10.1038/s41598-021-03646-5)
Supplement: Supplementary file 1 — Supplementary Information. [file 41598_2021_3646_MOESM1_ESM.pdf]

Supplementary Information to the manuscript: Intra-urban differentials of fetal mortality in clusters of social vulnerability in São Paulo Municipality, Brazil

Authors: Lays Janaina Prazeres Marques; Zilda Pereira da Silva; Bárbara Laisa Alves Moura; Rossana Pulcineli Vieira Francisco; Marcia Furquim de Almeida.

Supplementary Table S1. Changes in the Brazilian list of Avoidable Causes of Death (LBE) for application in stillbirths (proposed by the authors).

ORIGINAL LBE CLASSIFICATION	ADEQUACY BY THE AUTHORS
Category "Reducible with adequate healthcare to women in childbirth"	This category is not compatible with antepartum deaths, therefore, the causes classified in this group were transferred to "Reducible with adequate healthcare to women during pregnancy"
Category "Reducible with adequate healthcare to the fetus and the newborn"	The causes of this group were included in the "Reducible with adequate healthcare to women during pregnancy" for all types of death. Considering that death was intrauterine and that the fetus is an inseparable part of the mother, healthcare is entirely provided to the mother-fetus binomial. Thus, the category was renamed to "Reducible with adequate healthcare to women and their fetus during pregnancy".
Fetus and newborns affected by placenta and membrane complications (P02. 2, P02.3, P02.7, P02.8, P02.9), placenta praevia and placental abruption (P02.0 to P02. 1), and fetus and newborns affected by umbilical cord disorders (P02.4 to P02.6)	The antepartum deaths had the grouping of these causes in a single category "Fetus affected by complications of the placenta, cord, and membranes (P02)"
The causes of intrauterine hypoxia (P20) belonged to the group "Reducible with adequate healthcare to women in childbirth"	P20 codes were relocated to the "Ill-defined causes" group. Intrauterine hypoxia is often reported as a cause of death, but it is in fact the pathological pathway by which death occurs[9]
The causes of Down Syndrome (Q90) were considered "Reducible with adequate diagnostic and treatment actions".	The causes of Q90 were grouped into other congenital malformations defined as "Other causes (not clearly avoidable)".
The causes of "Other labor complications that affect the fetus and the newborn (P03.1, P03.5, P03.8)" and "Conditions that compromise the integument and thermal	These causes were considered as "Ill-defined causes of death", since these configure causes of death attributed to newborns.

regulation of the fetus and the newborn (P83.2)" belonged to the category "Reducible with adequate healthcare to women in childbirth".	
Deaths due to neonatal aspiration (P24) belonged to the group "Reducible with adequate healthcare for women in childbirth".	All deaths with a root cause identified as P24 were excluded from the study because they were infant deaths.
The causes considered "neonatal hemorrhage (P50 to P54, except P52)" were classified as "Reducible with adequate healthcare to newborns"	The causes of this group were readjusted, excluding P51 and P54 codes, exclusive to newborns. This category was renamed "Fetal blood loss" including only P50 and P53 codes, included in the category "Reducible with adequate attention to the woman and the fetus during pregnancy".
The causes related to "Other disorders originating in the perinatal period (P90 to P96.8, except P95)" were classified as "Reducible with adequate healthcare to the fetus and the newborn".	Codes P90 to P96.8 were allocated into the group of "Ill-defined causes of death". Codes assigned exclusively to newborns were excluded from this group: P90, P91, P92, P94, P96.1, P96.2, and P96.3. Thus, the following codes were kept in this category: P93, P96.4, P96.5, and P96.8, added to P96.9 (originally classified as ill-defined underlying cause).
Codes A50 belonged to the subgroup Congenital Syphilis.	The subgroup of codes A50 was grouped to other infections specific to the perinatal period (P35 to P39.9, except P35.0 and P35.3), being renamed to "congenital syphilis and other infections specific to the perinatal period".
The category "Ill-defined causes of death" consisted of codes R00 to R99 (except R95), P95, and P96.9.	The following codes were included in the category: P20, P21, P03.1, P03.5, P03.8, P83.2, P93, P96.4, P96.5, and P96.8.

Note: When only the 3-digit code is specified, it includes all 4-digit codes in the subcategory.

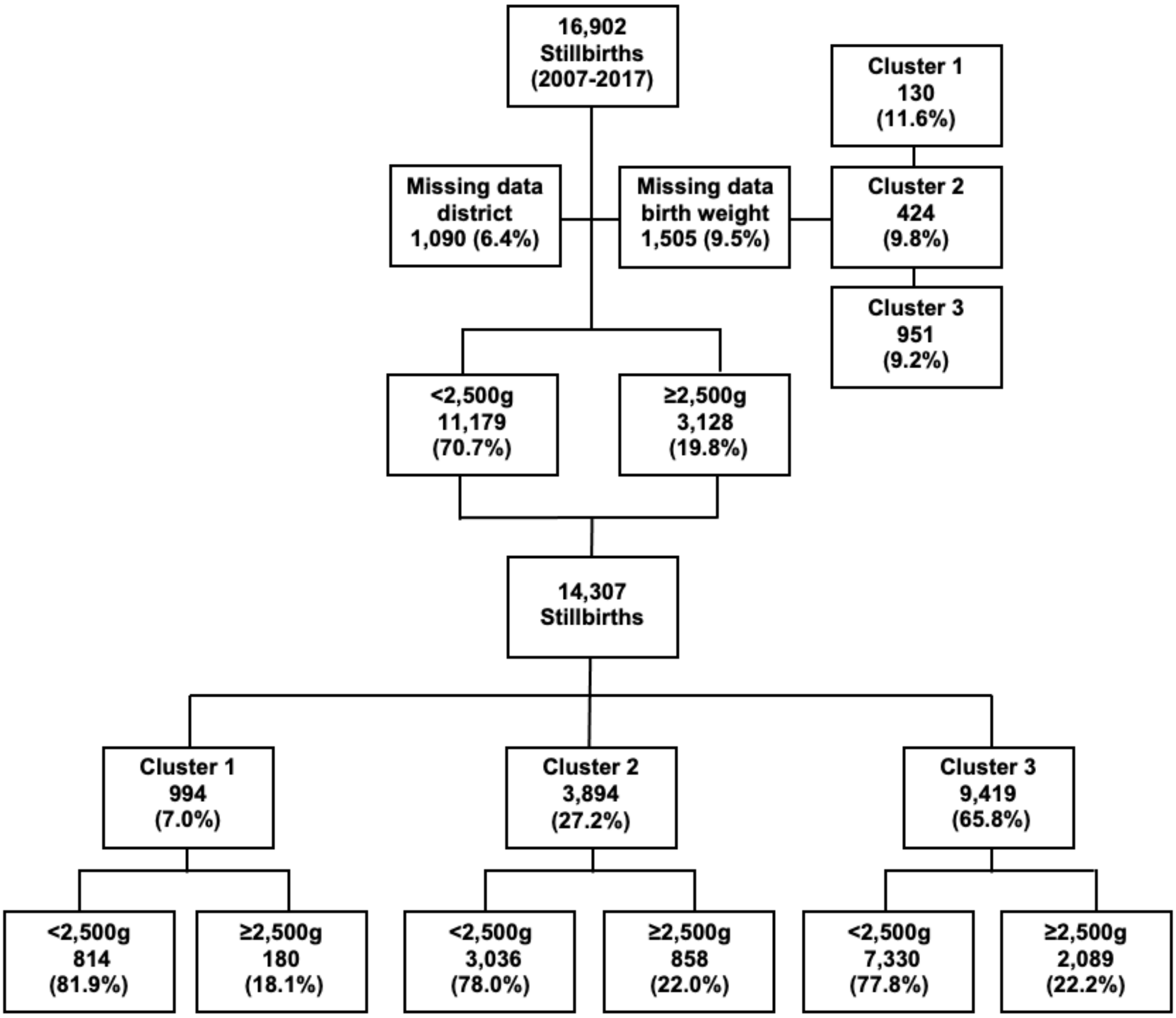

Supplementary Figure S1. Flowchart of the distribution of stillbirths by birth weight according to clusters of social vulnerability, São Paulo Municipality, 2007 to 2017.

Source: Mortality Information System.

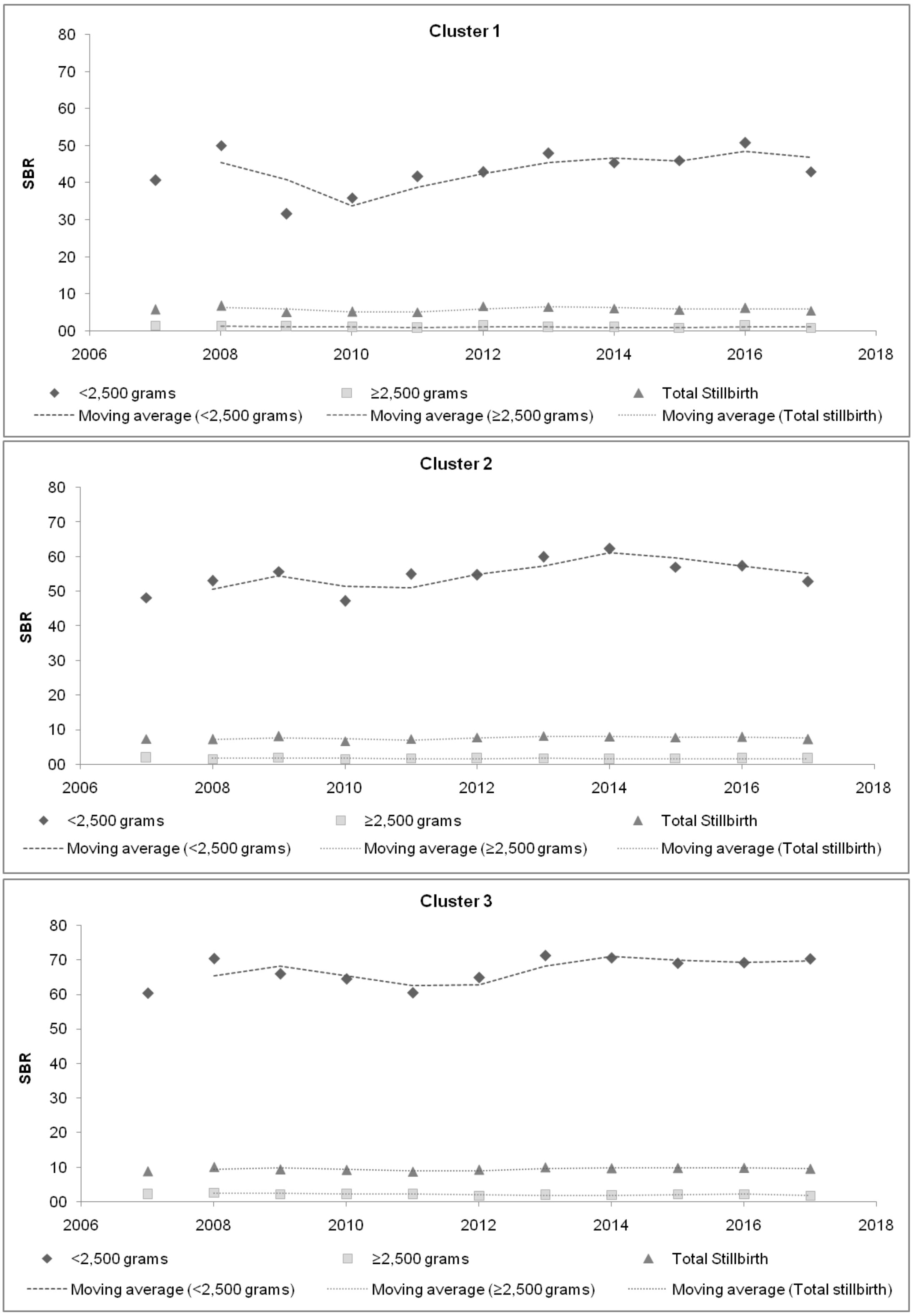

Supplementary Figure S2. Total stillbirth rate (per thousand births) and by birth weight, according to clusters of social vulnerability, São Paulo Municipality, 2007-2017.

Source: Mortality Information System and Live Birth Information System. Note: SBR – Stillbirth rate

Supplementary Table S2. Classification of avoidable stillbirth weighing <2,500g, according to clusters of social vulnerability, São Paulo Municipality, 2007-2017.

LBE groups and type of death	Cluster 1		Cluster 2		Cluster 3	
**Antepartum**	**n = 635**	**%**	**n = 2,265**	**%**	**n = 5,141**	**%**
**Avoidable causes**	**165**	**26.0**	**474**	**20.9**	**996**	**19.4**
**Reducible with adequate healthcare to women and their fetus during pregnancy**	**165**	**26.0**	**474**	**20.9**	**996**	**19.4**
Maternal conditions affecting the fetus (P00.1, P00. 2, P00.3, P00. 4, P00. 5, P00. 6, P00.8, P00.9)	42	23.9	142	28.2	326	30.9
Maternal complications during pregnancy affecting the fetus (P01.0, P01.1, P01. 2, P01.3, P01. 5, P01. 6, P01. 7, P01.8)	39	22.2	100	19.8	167	15.8
Fetus affected by complications of the placenta, cord, and membranes (P02.0, P02.1, P02.2, P02.3, P02.4, P02.5, P02.6, P02.7, P02.9)	73	41.5	202	40.1	458	43.4
Delayed fetal growth and fetal malnutrition (P05.9)	1	0.6	8	1.6	15	1.4
Disorders related to short-term pregnancy and low birth weight, not elsewhere classified (P07.0, P07.2)	2	1.1	7	1.4	15	1.4
Fetal blood loss (P50.3)	1	0.6	1	0.2	-	-
RH and ABO isoimmunization of the fetus (P55. 0)	-	-	1	0.2	1	0.1
Congenital syphilis and other infections specific to the perinatal period (A50.0, A50.2, P35.1, P35.9, P37.1, P39. 2, P39.9)	6	3.4	6	1.2	5	0.5
Transient endocrine and metabolic disorders specific to the fetus (P70.0, P70.1)	1	0.6	7	1.4	9	0.9
**Ill-defined causes of death**	**361**	**56.9**	**1,594**	**70.4**	**3,828**	**74.5**
Fetal death from unspecified cause (P95)	125	35.7	545	34.8	1,542	40.9
Other disorders originating in the perinatal period (P96.4, P96.8, P96.9)	3	0.9	10	0.6	14	0.4
Intrauterine hypoxia (P20.0, P20.1, P20.9)	222	63.4	1,009	64.5	2,213	58.7
Other complications of labor that affect the fetus (P03.1, P03.5, P03.8)	9	5.1	12	2.4	13	1.2
Disorders affecting the integument and thermal regulation of the fetus (P83.2)	2	1.1	18	3.6	46	4.4
**Other causes (not clearly avoidable)**	**109**	**17.2**	**197**	**8.7**	**317**	**6.2**
Congenital malformations (Q00 to Q99)	109	100.0	197	100.0	315	99.4
Other causes (D18.1, P29. 8)	-	-	-	-	2	0.6
**Intrapartum**	**n = 15**	**%**	**n = 50**	**%**	**n = 127**	**%**
**Avoidable causes**	**10**	**66.7**	**25**	**50.0**	**67**	**52.8**
**Reducible with adequate healthcare to women and their fetus during pregnancy**	**6**	**40.0**	**15**	**30.0**	**47**	**37.0**

Maternal conditions affecting the fetus (P00.1, P00. 2, P00.8)	1	16.7	2	13.3	11	23.4
Maternal complications during pregnancy affecting the fetus (P01.0, P01.1, P01.5, P01.7)	4	66.7	8	53.3	23	48.9
Fetus affected by complications of the placenta and membranes (P02.2, P02.7)	1	16.7	4	26.7	11	23.4
Delayed fetal growth and fetal malnutrition (P05.9)	-	-	1	6.7	-	-
Disorders related to short-term pregnancy and low birth weight, not elsewhere classified (P07.2)	-	-	-	-	2	4.3
**Reducible with adequate healthcare to women in childbirth**	**4**	**26.7**	**10**	**20.0**	**20**	**15.7**
Placenta praevia and placental abruption (P02. 0, P02.1)	2	50.0	5	50.0	9	45.0
Fetus affected by cord disorders (P02.5)	-	-	1	10.0	1	5.0
Other complications of labor that affect the fetus (P03.0, P03.5, P03.8)	2	50.0	4	40.0	10	50.0
**Ill-defined causes of death**	**3**	**20.0**	**17**	**34.0**	**43**	**33.9**
Fetal death from unspecified cause (P95)	2	66.7	6	35.3	21	48.8
Intrauterine hypoxia (P20.0, P20.1, P20.9)	1	33.3	11	64.7	22	51.2
**Other causes (not clearly avoidable)**	**2**	**13.3**	**8**	**16.0**	**17**	**13.4**
Congenital malformations (Q00 to Q99)	2	100.0	8	100.0	17	100.0
**Unknown**	**n = 164**	**%**	**n = 721**	**%**	**n = 2,062**	**%**
**Avoidable causes**	**15**	**9.1**	**49**	**6.8**	**140**	**6.8**
**Reducible with adequate healthcare to women and their fetus during pregnancy**	**9**	**5.5**	**36**	**5.0**	**106**	**5.1**
Fetus affected by complications of the placenta and membranes (P02.2, P02.3, P02.7, P02.8, P02.9)	3	33.3	5	12.8	18	15.4
Maternal conditions affecting the fetus (P00.0, P00. 1, P00.2, P00.3, P00.5, P00.6, P00.8, P04.4)	2	22.2	13	33.3	39	33.3
Maternal complications of pregnancy affecting the fetus (P01.0, P01.1, P01.2, P01.5, P01.9)	2	22.2	12	30.8	40	34.2
Disorders related to short-term pregnancy and low birth weight, not elsewhere classified (P07.2, P07.3)	2	22.2	3	7.7	5	4.3
Non-traumatic intracranial hemorrhage of the fetus (P52.9)	-	-	-	-	1	0.9
Congenital syphilis and other infections specific to the perinatal period (A50.2, P39.2, P39.9)	-	-	1	2.6	2	1.7
Fetal blood loss (P50.3, P54.9)	-	-	2	5.1	1	0.9
**Reducible with adequate healthcare to women in childbirth**	**6**	**3.7**	**13**	**1.8**	**34**	**1.6**
Placenta praevia and placental abruption (P02.0 to P02.1)	2	33.3	8	61.5	23	67.6
Fetus affected by umbilical cord disorders (P02.4 to P02.6)	1	16.7	2	15.4	6	17.6

Other complications of labor that affect the fetus (P03.1, P03.5, P03.8)	3	50.0	3	23.1	5	14.7
**Ill-defined causes of death**	**139**	**84.8**	**634**	**87.9**	**1,838**	**89.1**
Fetal death from unspecified cause (P95)	94	67.6	425	67.4	1,204	65.9
Intrauterine hypoxia (P20.0, P20.1, P20.9)	44	31.7	203	32.2	616	33.7
Other disorders originating in the perinatal period (P96.4)	1	0.7	3	0.7	7	0.6
Disorders affecting the integument and thermal regulation of the fetus (P83.2)	-	-	3	7.7	11	9.4
**Other causes (not clearly avoidable)**	**10**	**6.1**	**38**	**5.3**	**84**	**4.1**
Congenital malformations (Q00 to Q99)	10	100.0	38	100.0	84	100.0

Note: LBE - Brazilian List of Avoidable Causes of Death (*Lista Brasileira de Causas de Mortes Evitáveis)*

Supplementary Table S3. Classification of avoidable stillbirth weighing ≥2,500g, according to clusters of social vulnerability, São Paulo Municipality, 2007-2017.

LBE groups and type of death	Cluster 1		Cluster 2		Cluster 3	
**Antepartum**	n = 134	%	n = 636	%	n = 1,442	%
**Avoidable causes**	**38**	**28.4**	**135**	**21.2**	**227**	**15.7**
**Reducible with adequate healthcare to women and their fetus during pregnancy**	**38**	**28.4**	**135**	**21.2**	**227**	**15.7**
Maternal conditions affecting the fetus (P00.0, P00.2, P00.3, P00.4, P00.8, P00.9, P04.2)	5	13.2	19	13.8	52	21.5
Maternal complications of pregnancy affecting the fetus (P01.1, P01.2, P01.3, P01.5, P01.8, P01.9)	2	5.3	5	3.6	10	4.1
Fetus affected by complications of the placenta, cord, and membranes (P02.1, P02.2, P02.4, P02.5, P02.6, P02.7, P02.9)	27	71.1	83	60.1	142	58.7
Delayed fetal growth and fetal malnutrition (P05.9)	-	-	-	-	1	0.4
Fetal blood loss (P50.0, P50.9, P54.8)	-	-	3	2.2	1	0.4
Non-traumatic intracranial hemorrhage of the fetus (P52.4, P52.9)	-	-	2	1.4	-	-
Congenital syphilis and other infections specific to the perinatal period (P39.2)	-	-	1	0.7	-	-
Transient endocrine and metabolic disorders specific to the fetus (P70.0, P70.1)	4	10.5	21	15.2	21	8.7
Disorders related to prolonged pregnancy and high birth weight (P08.1)	-		1	0.7	-	-
**Ill-defined causes of death**	**89**	**66.4**	**475**	**74.7**	**1,166**	**80.9**
Fetal death from unspecified cause (P95)	25	28.1	161	34.1	432	37.5
Other disorders originating in the perinatal period (P96.4, P96.9)	-	-	2	0.4	2	0.2
Intrauterine hypoxia (P20.0, P20.1, P20.9)	64	71.9	309	65.5	717	62.3
Disorders affecting the integument and thermal regulation of the fetus (P83.2)	-	-	1	0.7	6	2.5
Other complications of labor that affect the fetus (P03.1, P03.8)	-	-	2	1.4	9	3.7
**Other causes (not clearly avoidable)**	**7**	**5.2**	**26**	**3.8**	**49**	**3.4**
Congenital malformations (Q00 to Q99)	7	100.0	26	100.0	49	100.0
**Intrapartum**	n = 4	%	n = 11	%	n = 33	%
**Avoidable causes**	**2**	**50.0**	**2**	**18.2**	**20**	**60.6**
**Reducible with adequate healthcare to women and their fetus during pregnancy**	**1**	**25.0**	**2**	**18.2**	**8**	**24.2**
Fetus affected by complications of the placenta and membranes (P02.2, P02.3, P02.7, P02.8, P02.9)	1	100.0	1	50.0	1	12.5
Maternal conditions affecting the fetus (P00.0, P00.1, P00.3)	-	-	1	50.0	4	50.0
Maternal complications during pregnancy affecting the fetus (P01.1, P01.7)	-	-	-	-	3	37.5

**Reducible with adequate healthcare to women in childbirth**	**1**	**25.0**	**-**	**-**	**12**	**36.4**
Placenta praevia and placental abruption (P02.0 to P02.1)	-	-	-	-	6	50.0
Fetus affected by umbilical cord disorders (P02.4 to P02.6)	1	100.0	-	-	3	25.0
Other complications of labor that affect newborns (P03.8)	-	-	-	-	1	8.3
**Ill-defined causes of death**	**1**	**25.0**	**8**	**72.7**	**12**	**36.4**
Fetal death from unspecified cause (P95)	1	100.0	2	25.0	5	41.7
Intrauterine hypoxia (P20.0, P20.9)	-	-	6	75.0	9	75.0
**Other causes (not clearly avoidable)**	**1**	**25.0**	**1**	**9.1**	**1**	**3.0**
Congenital malformations (Q00.0, Q97.7)	1	100.0	1	100.0	1	100.0
**Unknown**	**n = 42**	**%**	**n = 211**	**%**	**n = 614**	**%**
**Avoidable causes**	**3**	**7.1**	**13**	**6.2**	**29**	**4.7**
**Reducible with adequate healthcare to women and their fetus during pregnancy**	**1**	**2.4**	**3**	**1.4**	**14**	**2.3**
Maternal disorders affecting the fetus (P00.0)	1	100.0	2	50.0	6	1.0
Maternal pregnancy complications affecting the fetus (P01.5)	-	-	-	-	1	0.2
Fetus affected by complications of the placenta and membranes (P02.2)	-	-	-	-	6	1.0
Transient endocrine and metabolic disorders specific to the fetus (P70.0, P70.1)	-	-	1	25.0	1	0.2
**Reducible with adequate healthcare to women in childbirth**	**2**	**4.8**	**10**	**4.7**	**15**	**2.4**
Placenta praevia and placental abruption (P02.1)	-	-	5	50.0	7	46.7
Fetus affected by cord disorders (P02.5)	1	50.0	4	40.0	4	26.7
Other complications of labor that affect the fetus (P03.3, P03.8)	-	-	1	10.0	2	13.3
Disorders related to prolonged pregnancy and high birth weight (P08.1)	1	50.0	-	-	1	6.7
Birth trauma (P10.9)	-	-	-	-	1	6.7
**Ill-defined causes of death**	**37**	**88.1**	**192**	**91.0**	**560**	**91.2**
Fetal death from unspecified cause (P95)	23	62.2	119	62.3	350	62.7
Intrauterine hypoxia (P20. 0, P20.9)	14	37.8	70	36.6	208	37.3
Other disorders originating in the perinatal period (P96.4)	-	-	1	0.5	-	-
Disorders affecting the integument and thermal regulation of the fetus (P83.2)	-	-	1	0.5	2	0.3
Abnormal symptoms, signs, and findings from clinical and laboratory tests not elsewhere classified (R99)	-	-	1	0.5	-	-
**Other causes (not clearly avoidable)**	**2**	**4.8**	**6**	**2.8**	**25**	**4.1**
Congenital malformations (Q00 to Q99)	2	100.0	6	100.0	24	96.0
Other causes (P29.9)	-	-	-	-	1	4.0

Note: LBE - Brazilian List of Avoidable Causes of Death (*Lista Brasileira de Causas de Mortes Evitáveis)*
